# MicroRNA-486-3p functions as a tumor suppressor in oral cancer by targeting DDR1

**DOI:** 10.1186/s13046-019-1283-z

**Published:** 2019-06-28

**Authors:** Sung-Tau Chou, Hsuan-Yu Peng, Kuan-Chi Mo, Yuan-Ming Hsu, Guan-Hsun Wu, Jenn-Ren Hsiao, Su-Fang Lin, Horng-Dar Wang, Shine-Gwo Shiah

**Affiliations:** 1 National Institute of Cancer Research, National Health Research Institutes, No. 35 Keyan Road, Zhunan Town, Miaoli County 35053 Taiwan; 2 Institute of Biotechnology, National Tsing Hua University, Hsinchu, Taiwan; 3 Department of Otolaryngology, Head and Neck Collaborative Oncology Group, National Cheng Kung University Hospital, College of Medicine, National Cheng Kung University, Tainan, Taiwan; 4 Program in Environmental and Occupational Medicine, Kaohsiung Medical University, Kaohsiung, Taiwan

**Keywords:** miR-486-3p, DDR1, ANK1, DNA methylation, Oral cancer, OSCC

## Abstract

**Background:**

Discoidin domain receptor-1 (DDR1) tyrosine kinase is highly expressed in a variety of human cancers and involved in various steps of tumorigenesis. However, the precise mechanisms underlying the abnormal expression of DDR1 in oral squamous cell carcinoma (OSCC) has not been well investigated.

**Methods:**

The expression of DDR1 on OSCC patients was determine by quantitative real-time PCR (qRT-PCR) and immunohistochemistry. Specific targeting by miRNAs was determined by software prediction, luciferase reporter assay, and correlation with target protein expression. The functions of miR-486-3p and DDR1 were accessed by MTT and Annexin V analyses using gain- and loss-of-function approaches. Chromatin immunoprecipitation (ChIP) and methylation specific PCR (MSP) were performed to explore the molecular mechanisms by arecoline treatment.

**Results:**

Here, we reported that DDR1 was significantly upregulated in OSCC tissues and its levels were inversely correlated with miR-486-3p expression. The experimental results in vitro confirmed that miR-486-3p decreased DDR1 expression by targeting the 3′-UTR of DDR1 mRNA. Overexpression of miR-486-3p led to growth inhibition and apoptosis induction with a similar function by knockdown of DDR1. Aberrant methylation of *ANK1* promoter was a highly prevalent in OSCC and contributes to oral carcinogenesis by epigenetic silencing of ANK1 and miR-486-3p. We found that miR-486-3p can be transcriptionally co-regulated with its host gene *ANK1* through epigenetic repression. DNA methylation inhibitor treatment re-expressed *ANK1* and miR-486-3p. Importantly, arecoline, a major betel nut alkaloid, recruited DNMT3B binding to *ANK1* promoter for DNA methylation and then attenuated the expression of miR-486-3p in OSCC.

**Conclusion:**

This study was the first to demonstrate that betel nut alkaloid may recruit DNMT3B to regulate miR-486-3p/DDR1 axis in oral cancer andmiR-486-3p and DDR1 may serve as potential therapeutic targets of oral cancer.

**Electronic supplementary material:**

The online version of this article (10.1186/s13046-019-1283-z) contains supplementary material, which is available to authorized users.

## Background

Oral cancer, predominantly oral squamous cell carcinoma (OSCC), is one of highly prevalent and lethal cancers in the world, its incidence and mortality have significantly increased over the past decade [1]. Although the advances in surgery, radiotherapy, chemotherapy and targeted therapy have tremendously improved, the 5-year overall survival of oral cancer patients has not significantly improved in past 20 years [2–4]. In Taiwan, alcohol, betel-nut and cigarette consumption are the most common risk factors for the development of oral cancer [5]. Among these factors, betel quid chewing has been recognized as the major contributing factor for oral cancer incidence and mortality rate [6]. Therefore, understanding the molecular characteristics of betel quid chewing-associated oral cancer and finding the promising therapeutic targets have potential significance for informing prognosis and improving the clinical strategies of oral cancer.

Discoidin domain receptor-1 (DDR1) is a member of a subfamily of Discoidin domain receptors. DDR1 expresses multiple isoforms (DDR1a-e) generated through alternative splicing. DDR1a-c encode full-length and functional receptors and DDR1d-e encode truncated or inactive kinases [7]. This subfamily receptor has a similar structural framework, which consists of the N-terminal discoidin homology (DS) domains, extracellular juxtamembrane (JM), single transmembrane (TM) domain, unusually large cytosolic JM domain, tyrosine kinase domain and a short C-terminal tail [7, 8]. DDR1 can be activated by both fibrillary type collagens (I-III) and basement membrane collagen (IV) [9–11]. This receptor-ligand binding exhibits an atypical status with a slow and sustained phosphorylation. Upon activation, DDR1 undergoes autophosphorylation at multiple tyrosine residues located at the cytoplasmic domain and transmit signals into cells [12]. Aberrant expression and activation of DDR1 have been reported in several human cancers, such aslung cancer [13, 14], breast cancer [15], brain cancer [16, 17], oral cancer [18, 19] and liver cancer [20]. Like many other receptor tyrosine kinases, the dysregulated DDR1 display crucial roles in tumor initiation and progression, such as survival, proliferation, adhesion, migration, metastasis, epithelial-mesenchymal transition (EMT) and drug resistance [7].

Several mechanisms have been reported to cause abnormal expression of DDR1 in cancer, such as somatic mutations [21], transcriptional regulation [22] and microRNA (miRNA) regulation [20]. As endogenous small noncoding RNAs, miRNAs function through interacting with the 3′-untranslated region (3′-UTR) of targeted mRNAs, which cause inhibition of translation or even mRNA degradation [23, 24]. Accumulating evidence suggests that miRNAs can act as either oncogenes or tumor suppressors by affecting proliferative signaling, apoptosis, immortality, angiogenesis, invasion and metastasis [25–27]. Specifically, miR-199a-5p has been reported to target DDR1 in hepatocellular carcinoma [20], acute myeloid leukemia [28], colorectal cancer [29] and cutaneous squamous cell carcinoma [30]. Moreover, decreased expression of miR-199a-5p contributes to increased cell migration, invasion and tumorigenic capabilities through upregulating DDR1 expression [20, 29]. However, the expression level of miR-199a-5p is not aberrant in OSCC tissues compared with corresponding non-tumor tissues in our cohort of OSCC patients [31]. Obviously, there should be other miRNAs involving the DDR1 regulationin OSCC. Therefore, in the present study we sought to explore a miRNA-mediated molecular pathway leading to DDR1 dysfunctionin oral cancer.

In this study, we found that miR-483-5p is revealed as an upstream regulator of DDR1 which confers the cell proliferation and anti-apoptosis in OSCC. Further experiments demonstrated that miR-486-3p was directly bound to the 3′-UTR of DDR1 and downregulate DDR1. In OSCC tissues, miR-483-5p expression was downregulated while DDR1 expression was upregulated, and a negative correlation was found between miR-486-5p and DDR1 expression. Moreover, miR-486-3p can be transcriptionally co-regulated with its host gene ANK1 through epigenetic mechanism. Specifically, arecoline, a major betel nut alkaloid, could recruit DNMT3B binding to ANK1 promoter and caused DNA methylation. Together, our study indicates that betel quid chewing may induce aberrant methylation in OSCC interrupts the inhibitory effect of miR-486-3p on DDR1 expression which consequently promotes the oncogenic activity. This finding highlights its potential clinical applications and providing a new information for the development of targeted molecular therapy of oral cancer.

## Methods

### Clinical samples and patient characteristics

Paired tumor specimens and their adjacent non-tumorous epithelia were obtained from primary OSCC patients who received curative surgery from 1999 to 2010 at the National Cheng Kung University Hospital (Tainan, Taiwan). Fresh frozen tissues were preserved in liquid nitrogen until use. The study protocol was reviewed and approved by the Institutional Human Experiment and Ethic Committee of the National Cheng Kung University Hospital (No: HR-97-100) and the National Health Research Institutes (No: EC1040409-E). These matched pairs of oral tumor/adjacent normal (T/N) tissues were grouped into two sets, i.e., a training set containing 40 samples for genome wide microarray study and a validation set containing 46 samplesfor DDR1 quantitative-PCR analysis. Otherwise, we also collected another 75 of OSCC patients to quantify the expression levels of DDR1 using quantitative-PCRfor Kaplan-Meier survival analyses. All the clinical information of these 3 different cohorts, were summarized and listed in Additional file 1: Table S1.

### Microarray profiling

Clinical profiling data of 40 primary OSCC frozen samples and corresponding matched normal samples were prepared as training group for microarray analysis. Gene expression profiling was performed using the whole-genome DASL HumanRef-8-v3 chip, miRNA expression profiling was performed using the Human-2v MicroRNA Expression BeadChips and methylation profiling was performed using the infinium WG methylation27 chip (Illumina, Inc.). Microarray data processing and analysis were done using Illumina BeadStudio software. Microarray Data are available in Gene Expression Ommibus (GEO) under accession number GSE37991 for gene expression, GSE45238 for miRNA expression and GSE38823 for methylation analysis.

### Immunohistochemistry (IHC)

OSCC tissue array (#OR601a, #OR2081, #HN811 and #BC34011, Biomax Inc., Rockville, MD) were deparaffnized using xylene and then rehydratedthrough an ethanol series. Antigens were retrieved by autoclaving he slides in Dako retrieval buffer. After cooling the slides to room temperature, they were incubated with primary DDR1 antibody (sc-532, Santa Cruz, CA, USA) at 4 °C overnight. Specific signals were then developed with LSAB^+^ kit (DakoCytomation, CA, USA) using diaminobenzidine as chromogen. Finally, the sections were counterstained with haematoxylin.

### Cell culture, vectors and reagents

OKF4/hTERT obtained from Rheinwald lab, and cultured in oral keratinocyte medium according to the manufacturer’s instructions. OEC-M1 and TW2.6 OSCC cell lines were routinely cultured as previously described [32]. All cells were guaranteed by morphology and growth characteristics, and cultured at 37 °C in a 5% CO_2_ atmosphere and maintained in 10% FBS (Kibbutz) within 3 months of resuscitation from the frozen stock, with fewer than 20 passages. Cells were treated with 5-Aza-dC (5 μM, A3656, Sigma), arecoline (100 μM, MERCK) or lentiviral infected with shRNA vectors as described in Additional file 1: Table S2.

### Protein extraction and western blotting analysis

Protein extraction and western blotting were performed as previously described [33]. Primary antibodies were rabbit anti-DDR1 (sc-532, 1:500, Santa Cruz, CA, USA), mouse anti-Bcl2 (sc-1097, 1:200, Santa Cruz, CA, USA), rabbit anti-PARP (#5625, 1:1000, Cell Signaling, USA), rabbit anti-Caspase-3 (#9662, 1:1000, Cell Signaling) and rabbit anti-GAPDH (GTX100118, 1:200000, GeneTexInc, Irvine, CA, USA). After washing, blots were incubated with appropriate secondary antibodies.

### RNA extraction, reverse transcription and quantitative-PCR (q-PCR)

Total RNAs were isolated using TRIzol (Ambion) based on the manufacturer’s protocol. For mRNA analysis, the cDNA was synthesized using random hexamer primers andSuperScript III reverse transcriptase (Invitrogen, Carlsbad,CA). For miRNA analysis, the cDNA was synthesized usingspecific stem-loop RT primers and TaqMan MicroRNA Reverse Transcription Kit (Applied Biosystems). q-PCR analysis was used to detectthe DDR1 and ANK1 using Maestro GreenEvaGreen q-PCR Master Mix (Maestrogen) and the expression level of miR-486-3p using QuantiTect SYBR Green PCR System (QIAGEN),respectively, according to the manufacturer’s instructions on the ABI StepOnePlus Real-time PCR system (Applied Biosystems). GAPDH and RUN44 were usedas the internal control. All reactions were run in triplicate and relative expression levels were calculated as 2^-△△CT^ after normalization with internal control. All primers used for this study are summarized in Additional file 1: Table S3.

### Targeting miRNAs prediction

Prediction of miRNAs that target DDR1 was performed by TargetScan Release 6.2 (http://www.targetscan.org) and microRNA.org (http://www.microrna.org/microrna/home.do). Otherwise, differentially expressed miRNAs that display 2-fold downregulation with a high concordance in OSCC tumors (GSE45238) were identified. Combining these approaches, putative miRNAs targeting DDR1 predicted by both two algorithms and microarray data wereaccepted and candidates were chosen based on experimental confirmation.

### Plasmid construction and luciferase reporter assay

The entire 3′-UTR of DDR1 fragment, containing target sequences of miR-337-3p and miR-486-3p, were PCR-amplified and cloned into the pmirGLO firefly luciferase-expressing vector (Promega, WI, USA) according to the manufacturer’s instructions. The miR-486-3p binding site mutation vectors were also constructed by using Site-Directed Mutagenesis Kit (Stratagene, La Jolla, CA), and all the constructs were verified by DNA sequencing. For luciferase reporter assay, luciferase reporter vectors together with miRNA-mimics (PM-337-3p or PM-486-3p) and negative control (NC) were transfected into HEK293,OEC-M1 and TW2.6 cells using Lipofectamine RNAiMAX Transfection Reagent (Thermo Fisher). After 48 h, luciferase activities were detected using the Dual Luciferase Reporter Assay System (Promega) on the Orion L luminometer (Berthold), according to the manufacturer’s protocol. Renilla luciferase served as the control reporter for normalization.

### Cell proliferation assay

Cell proliferation was measured using 3-[4,5-dimethylthiazol-2-yl]-2,5-diphenyl tetrazolium bromide (MTT, Sigma-Aldrich, St. Louis, MO, USA) as previously described [34]. The optical density (OD) at 550 nm was measured using a 96-well plate SpectraMax 250 reader (Molecular Devices, CA, USA). For colony formation assay, cells (150 cells/well for OEC-M1 and 300 cells/well for TW2.6) were seeded into 6-well plates and cultured for 10 days at 37 °C in culture hood. Colonies were fixed, stained with 1% crystal violet and counted under a microscope.

### Cell apoptosis assay

For cell apoptosis assay, cells were seeded in 6-well plates and transfected with miR-486-3p mimics (PM-486-3p) and negative control (NC). Double staining with FITC-conjugated annexin V and propidium iodide (PI) was performed as follows. Forty-eight hours post-transfection, the cells, including floating cells, were harvested, washed twice with 4 °C PBS and resuspended in binding buffer (10 mM HEPES/NaOH, 140 mMNaCl, 2 mMKCl). Annexin V was added for 15 min in the dark. Cells were then washed, centrifuged and resuspended in binding buffer. Before flow cytometric analysis, PI was added to each sample. Annexin V ^+^/PI^−^ cells were early apoptotic cells.

### Virus production and infection of target cells

The lentiviral backbone plasmids were transfected into the packaging cell line 293FT, along with pMD. G and pCMV△R8.91 plasmid, using the Polyjet transfection reagent (SignaGen Lab, Ijamesville, MA, USA). After 48 h incubation, the media containing lentivirus were harvested and centrifuged at 3000 rpm for 5 min. After 48 h incubation, the viral supernatants were transferred to the target cells in the presence of polybrene (8 μg/mL) at 37 °C for 6 h. Then, the cells were replaced with fresh growth media for another 48 h incubation until analysis.

### Methylation specific PCR (MSP)

Genomic DNA, extracted by using the DNA Extraction Kit (Qiagen), was bisulfite-modified by the EZ-DNA Methylation-Gold Kit (Zymo Research) according to the manufacturer’s instructions. The bisulfite reaction converted non-methylated cytosines to uracils and ultimately detected as thymidines after PCR amplification. The forward and reverse primers targeting methylated and unmethylated promoter region of the human *ANK1* gene are listed in Additional file 1: Table S**3**. The PCR products amplified by the methylated (M) and unmethylated (U) primers were 79 base pair and 84 base pair, respectively. The MSP products were visualized by electrophoresis with 2% agarose gel containing ethidium bromide.

### Chromatin immunoprecipitation (ChIP) assay

ChIP assay was performed based on previous described [35]. OKF4/hTERT cells treated with 100 μM of arecoline for 5 days and then fixed with formaldehyde for cross-link chromatin associated proteins to genomic DNA, lysed and sonicated to generate DNA fragments between 200 to 1000 base pair (confirmed by agarose gel electrophoresis). Then, the cell lysate were subjected to immunoprecipitation overnight by DNMT3A (sc-20,701, Santa Cruz, CA, USA) or DNMT3B (ab2851, Abcam) antibodies and consequently for PCR assay. Primers used for this study are summarized in Additional file 1: Table S**3**.

### Statistical analysis

Group differences were analyzed by the two-tailed Student *t* test. All statistical analysis and graph presentation were performed using GraphPad Prism 5 Software Ver. 5.01 (GraphPad, San Diego, CA). A value of *P* < 0.05 was considered as statistically significant.

## Results

### DDR1 is upregulated in oral cancer patients and cell lines

**Fig. 1:**
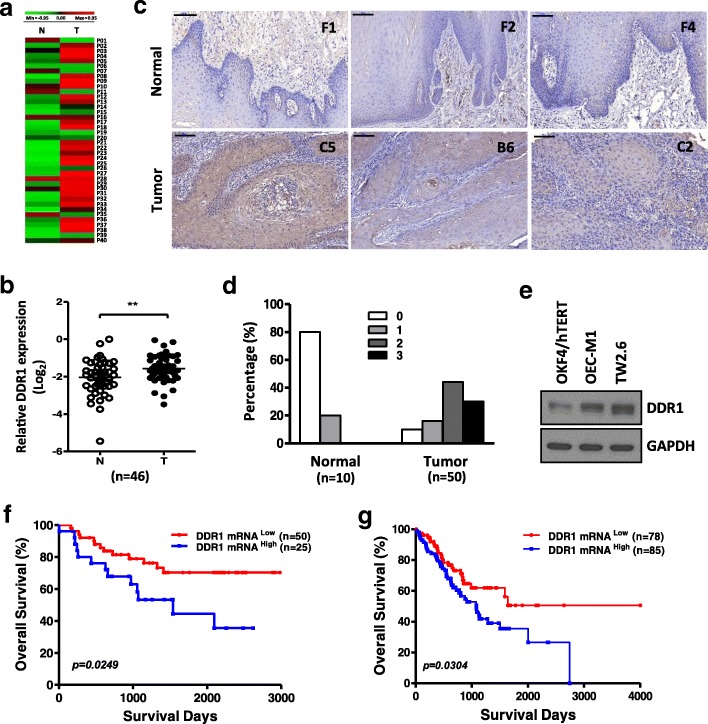
DDR1 is up-regulated in oral cancer patients and cell lines **a:** Heat map of DDR1 expression in 40 OSCC tissue pairs (GSE37991). Red, overexpression; green, downexpression. **b:** Validation of DDR1 expression by qRT-PCR in another 46 of OSCC tumors (T) compared with their own adjacent normal tissues (N). DDR1 expression levels are expressed as the log_2_ ratios. All data are represented as mean ± SD; **p < 0.01.**c:**Immunohistochemical analysis of DDR1 in OSCC tissue array (#OR601a, Biomax), containing 50 cases of OSCC and 10 cases of adjacent normal tissues. Representative samples showing different levels of DDR1 selected from normal tissues (F1, F2 and F4) and tumor tissues (C5, B6 and C2), respectively. The scale bars in the right upper corners of all photos are equivalent to 100 μm. **d:** Bar charts show the percentage of DDR1 staining score for the oral specimens in the tissue microarray (#OR601a, Biomax). The DDR1 staining intensity was scored as follows: 0, negative; 1, week;2, mediate and 3, strong.**e:** Western blot analysis of DDR1 expression in normal oral keratinocyte (OKF4/hTERT) and OSCC cell lines (OEC-M1 and TW2.6). Glyceraldehyde-3-phosphate dehydrogenase (GAPDH) was used as an internal control.f: Kaplan-Meier survival analyses of DDR1 in overall survivalsurvival of patients with oral cancer was determined in our cohort (*n* = 75). g: Kaplan-Meier survival analyses of DDR1 in overall survival of patients with OSCC cancer was determined in TCGA’s cohort (*n* = 163)

To examine whether DDR1 plays an important role in OSCC, we analyzed the DDR1 expression levels from our previous genome-wide cDNA microarray profiles of 40 pairwise OSCC patients [31]. The data showed that DDR1 exhibited higher expression in most OSCC patients (82.5%) in the tumour tissues than the matched normal tissues (Fig. 1a). Further clinical analysis showed that high expression of DDR1 was statistically associated with lymph node metastasis, perineural invasion and lymphangiogenesis (*p* < 0.05) (Table S**4**). Using quantitative RT-PCR, the significantly upregulated DDR1 expression levels in tumorswere validated in another 46 of OSCC tissue samples (*p* < 0.01; Fig. 1b). Additionally, immunohistochemical analysis of DDR1 in the representative tissue array of OSCC patients showed stronger positive staining in cancer tissues compared with non-cancerous tissues (Fig. 1c, d). And this result has also been confirmed by another tissue array cohort which indicate that DDR1 were highly expressed in OSCC specimens, and their overexpression levels were associated with tumor grades (*p* < 0.05) (Additional file 2: Figure S1). In addition, western blotting analysis supported that DDR1 displayed higher expression levels in OSCC cell lines when compared with the transformed normal human keratinocyte (OKF4/hTERT, Fig. 1e). Furthermore, Kaplan-Meier analysis revealed that DDR1 expression was correlated with poor overall survival in oral cancer patients (*n* = 75) in our cohort (*p* = 0.0249; Fig. 1f) and in TCGA’s OSCC cohort (*n* = 163) (*p* = 0.0304; Fig. 1g). In addition to univariate model, we have run multivariate analysis considering treatment and other factors as covariates (Table S5–7) to exclude the impact of different treatments on the prognosis. Maybe the sample size is too small, we didn’t observe the significance of DDR1 expression in our training cohort (*n* = 40) and validation cohort (*n* = 46). However, as shown in Table S7 (n = 75), the expression of DDR1, when stratified into high and low groups, was still significantly associated with overall survival of OSCC patients, indicating DDR1 is a prognostic marker independent of treatment modality.

### Knockdown of DDR1 inhibits proliferation and induces apoptosis in OSCC cells

**Fig. 2:**
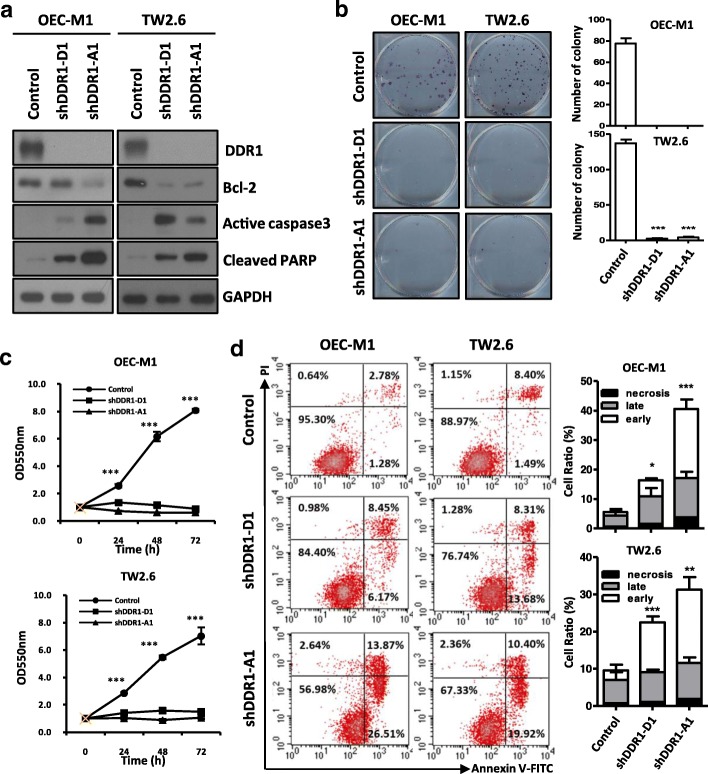
Knockdown of DDR1 causes an anti-tumor effect on oral cancer cells **a:** Western blot analysis of Bcl-2, cleaved caspase 3 and PARP in OEC-M1 and TW2.6 cells following DDR1 knockdown (shDDR1-D1 and –A1) for 48 h. GAPDH was used as an internal control. **b:** Colony formation assay after knockdown of DDR1 in OEC-M1 and TW2.6 cells for 10 days (*left*). The mean number of colonies for each well was determined from three independent assays (*right*). **c:** Growth rates of OEC-M1 and TW2.6 cells measured by MTT assay after DDR1 knockdown. **d:** *Left*: Dot plot for flow cytometric analysis of apoptotic cells after DDR1 knockdown (shDDR1-D1 and –A1) in OEC-M1 and TW2.6 cells for 48 h. Annexin V^−^/PI^−^: viable cells (lower left quadrant), Annexin V^−^/PI^+^: necroticcells (upper left quadrant), Annexin V^+^/PI^−^: early apoptotic cells (lower rightquadrant), Annexin V^+^/PI^+^: late apoptotic cells (upper right quadrant). *Right*: Bar graph quantifying the percentage of, early apoptotic (early), late apoptotic (late) and necrotic cells (necrosis) according to DDR1 knockdown (shDDR1-D1 and –A1). All data are presented as mean ± SD; *p < 0.05; **p < 0.01; ***p < 0.001 versus non-targeting shRNA plasmid (control)

To investigate thefunction of DDR1 in OSCC cells, knockdown of DDR1 was performed by infecting OEC-M1 and TW2.6 cells with shDDR1 lentiviral particles and examine the effects on colony formation and cell proliferation. We used two different shRNA sequences (shDDR1-A1 and shDDR1-D1) to knockdown DDR1 expression (Fig. 2a). As shown in Fig. 2b and c, DDR1 knockdown suppressed colony formation and cell proliferation compared with control cells in both OSCC cell lines (*P* < 0.0001). In addition, knockdown of DDR1 significantly decreased Bcl-2 protein level and induced caspase-3activation and PARP cleavage in OSCC cells (Fig. 2a). Moreover, annexin V/PI double staining assay of shDDR1-infected OSCC cells significantly increases the population of early apoptotic and late apoptotic cells, confirming the apoptotic effect of shDDR1 on OSCC cells (Fig. 2d). Collectively, these data indicate that DDR1 may influence the proliferation and apoptosis properties in OSCC cells.

### MiR-486-3p targets DDR1 in oral cancer

**Fig. 3:**
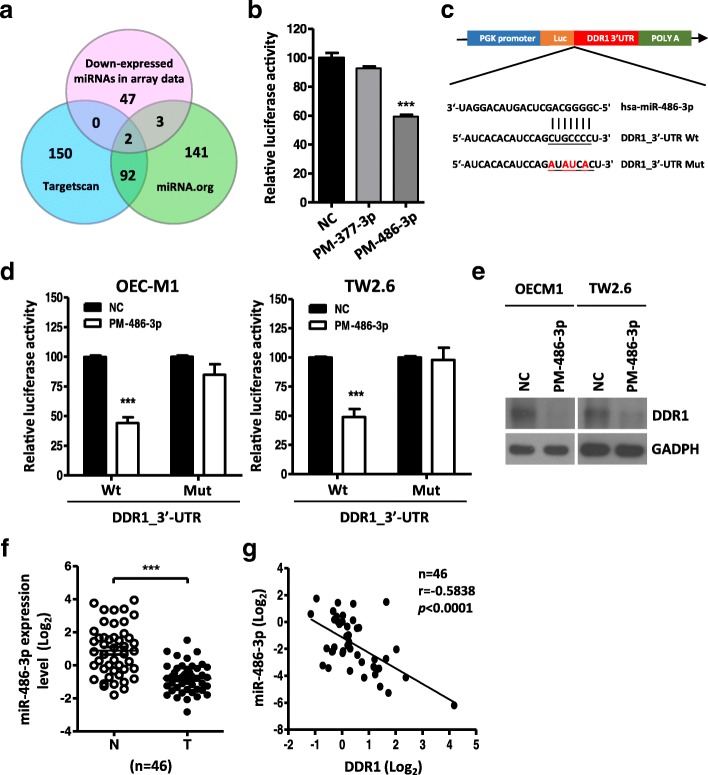
DDR1 is a direct target of miR-486-3p **a:** Venn diagram of predicted miRNA which targets DDR1 3′-UTR using two independent algorithms (miRNA.org and Targetscan) combined with our patients miRNA array data (GSE45238). **b:** The luciferase reporter assays with pmirGLO firefly luciferase constructs containing full length of DDR1 3′-UTR in HEK293 cells. The relative luciferase activity of each sample is measured at 48 h after transfection with 20 nM of miRNA mimics (PM-377-3p or PM-486-3p) and normalized to Renilla luciferase activity. The data are represented as mean ± SD; ***p < 0.001 versus control mimics (NC). **c:** Schematic representation of the putative miR-486-3p binding sequence in the 3′-UTR of DDR1 with wild-type form (DDR1_3′-UTR Wt) and mutant form (DDR1_3’-UTR Mut). The mutated nucleotides are labeled with red color.**d:** The effect of miRNA mimics (PM-486-3p, 20 nM) on the luciferase activities of the constructs containing the wild-type or mutant-type 3’-UTR in OEC-M1 (*left*) and TW2.6 (*right*) cells. The relative luciferase activity of each sampleis measured at 48 h after transfection and normalized to Renilla luciferase activity. The data are represented as mean ± SD; ***p < 0.001 versus control mimics (NC). **e:** Western blot analysis of DDR1 in OEC-M1 and TW2.6 cells following miR-486-3p mimics (PM-486-3p, 20 nM) transfection for 48 h. **f:** Validation of miR-486-3p expression by qRT-PCR in 46 of OSCC tumors (T) compared with their own adjacent normal tissues (N). DDR1 expression levels are expressed as the log_2_ ratios. All data are represented as mean ± SD; ***p < 0.001. **g:** Correlation analysis of miR-486-3p and DDR1 inhuman OSCC patients (*n* = 46) by qRT-PCR analysis

To test whether DDR1 was targeted by miRNAs, we used targeting algorithms (TargetScan and miRNA.org) combined with OSCC patients’ miRNA microarray data [31] to search for putative miRNAs that might bind toDDR1 mRNA. Based on the computational algorithm, we found miR-337-3p and miR-486-3p were downregulated in OSCC tissues compared with their matched adjacent non-cancerous tissues [31] and were selected for the further study (Fig. 3a). To determine whether miR-337-3p and miR-486-3p directly target DDR1 3′-UTR, a luciferase reporter assay was performed in HEK293 cells. We found that only miR-486-3p, but not miR-337-3p, transfected in HEK293 cells can significantly reduce the luciferaseactivity relative to the scrambled control (NC)(Fig. 3b). The luciferase activity of the reporter that contained DDR1 3′-UTR was significantly suppressed by miR-486-3p mimic (PM) transfection, but the activity of the reporter that contained DDR1 mutant 3′-UTR had no significant change. (Fig. 3c, d). These results suggested that miR-486-3p may suppress DDR1 expression by targeting the putative binding site in the DDR1 3′-UTR. To consolidate our findings, we performed western blotting to measure the DDR1 protein levels in OSCC cells. The data showed that DDR1 protein level was dramatically down-regulated by the transfection of miR-486-3p mimics (PM-486-3p) in OEC-M1 and TW2.6 (Fig. 3e). Furthermore, the miR-486-3p expression levels were validated in 46 OSCC patients by real-time PCR and were significantly decreased in OSCC tumors compared with their corresponding normal samples (*P* < 0.0001; Fig. 3f). Remarkably, we also correlated the expression level of miR-486-3p to DDR1 mRNA and found a significant inverse correlation between miR-486-3p and DDR1 (r = − 0.5838, *p* < 0.0001; Fig. 3g). These data suggested that DDR1 as a direct target of miR-486-3p and repression of miR-486-3p in OSCC cells directly increased expression of DDR1.

### Overexpression of miR-486-3p also inhibits proliferation and activates apoptosis in OSCC cells

**Fig. 4:**
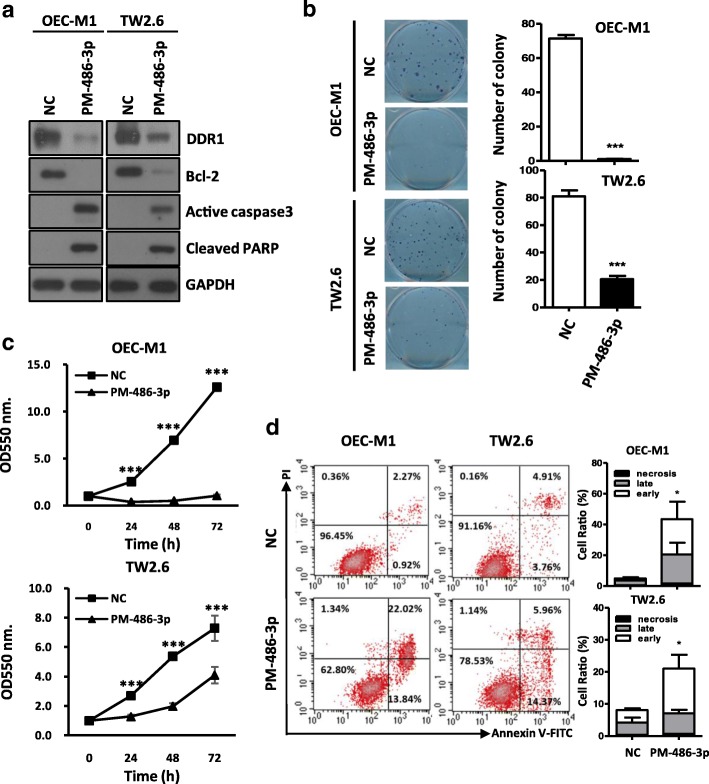
The effects of miR-486-3p overexpression on OSCC cells **a:** Western blot analysis of DDR1, Bcl-2, cleaved caspase 3 and PARP in OEC-M1 and TW2.6 cells following miR-486-3p (PM-486-3p) overexpression for 48 h. GAPDH was used as an internal control. **b:** Colony formation assay after miR-486-3p (PM-486-3p) transfection in OEC-M1 and TW2.6 cells for 10 days (*left*). The mean number of colonies for each well was determined from three independent assays (*right*). **c:** Growth rates of OEC-M1 and TW2.6 cells measured by MTT assay after miR-486-3p (PM-486-3p) transfection. **d:** *Left*: Dot plot for flow cytometric analysis of apoptotic cells after miR-486-3p (PM-486-3p) transfection in OEC-M1 and TW2.6 cells for 48 h. Annexin V^−^/PI^−^: viable cells (lower left quadrant), Annexin V^−^/PI^+^: necroticcells (upper left quadrant), Annexin V^+^/PI^−^: early apoptotic cells (lower rightquadrant), Annexin V^+^/PI^+^: late apoptotic cells (upper right quadrant). *Right*: Bar graph quantifying the percentage of, early apoptotic (early), late apoptotic (late) and necrotic cells (necrosis) according to miR-486-3p (PM-486-3p) transfection. All data are presented as mean ± SD; *p < 0.05; **p < 0.01; ***p < 0.001 versus negative control (NC)

The previous results have implicated DDR1 in cell proliferation and apoptosis in oral cancer cells. Therefore, we assessed the effects of miR-486-3p in the proliferation and apoptosis to determine whether overexpression of miR-486-3p mimics the biological functionsof DDR1 knockdown in OSCC cells. The DDR1 protein levels were significantly downregulated after ectopic expression of miR-486-3p (Fig. 4a). Ectopic overexpression of miR-486-3p decreased colony-forming ability in clonogenic proliferation assays (Fig. 4b) and reduced the growth rates of OSCC cells in MTT assays (Fig. 4c). Furthermore, ectopic overexpression of miR-486-3p reduced Bcl-2 expression, and induced active caspase-3 and cleaved PARP expression (Fig. 4a), indicating an apoptosis induction. The effect of miR-486-3p on apoptosis was further verified by annexin V/PI double staining assay. The results showed that the significantly higher apoptosis cells (early apoptotic and late apoptotic cells) were detected by miR-486-3p overexpression in OSCC cells (Fig. 4d). Taken together, these results suggest that ectopic expression of miR-486-3p can inhibit cell growth and activate apoptosis though repressing DDR1 expression.

### ANK1 methylation status effect miR-486-3p expression in OSCC cells

**Fig. 5:**
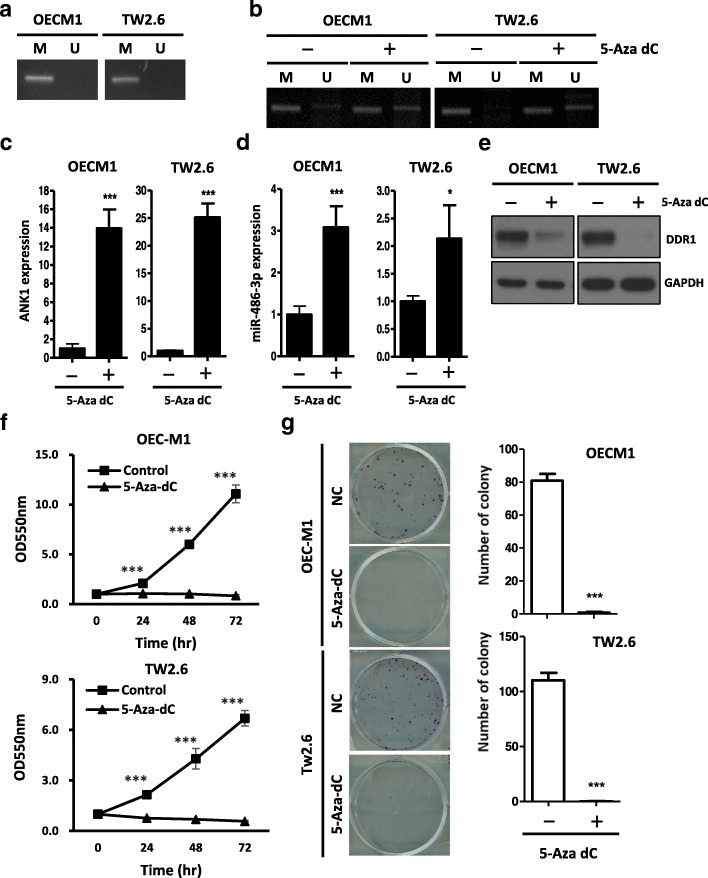
Methylation status of ANK1 promoter in oral cancer **a:** Methylation specific PCR of ANK1 promoter region in OEC-M1 and TW2.6 cells. Primers arespecific for unmethylated (U) or methylated (M) DNA.**b:** OEC-M1 and TW2.6 cells were treated without or with 5-Aza-dC (5 μM) for 5 days. Methylation status of ANK1 promoter region were measured by methylation specific PCR. **c:** qRT-PCR analysis of ANK1 expression after 5-aza-dC treatment in OEC-M1 and TW2.6 cells (mean ± SD; ***p < 0.001). **d:** qRT-PCR analysis of miR-486-3p expression after5-aza-dC treatment in OEC-M1 and TW2.6 cells (mean ± SD; *p < 0.05; ***p < 0.001). **e:** Western blot analysis of DDR1 protein after 5-Aza-dC (5 μM) treatment for 5 days. GAPDH was used as an internal control. **f:** Growth curves of OEC-M1 and TW2.6 cells measured by MTT assay after 5-Aza-dC (5 μM) for indicated time (mean ± SD; ***p < 0.001). **g:** *Left*: Colony formation assay after 5-Aza-dC (5 μM) treatment in OEC-M1 and TW2.6 cells for 10 days. *Right*: The mean number of colonies for each well was determined from three independent assays. All data are presented as mean ± SD; *p < 0.05; **p < 0.01; ***p < 0.001 versus control (NC)

Since miR-486-3p has been reported as co-expression with its host gene ANK1, and ANK1 expression level was reported to be regulated through promoter methylation [36]. To determine whether ANK1 promoter is hypermethylated in OSCC cells, we examined the methylation status of OSCC cell lines usingmethylation specific PCR (MSP). MSP analysis revealed that ANK1 promoter region was highly methylated in OEC-M1 and TW2.6 cells (Fig. 5a). Treatment with 5-Aza-dC, a DNA methylation inhibitor, decreased the methylation level of ANK1 promoter and elevated the expression of ANK1 mRNA (Fig. 5b, c). Simultaneously, we foundthatthe expression of miR-486-3p significantly increasedwhile DDR1 expression were downregulated after 5-Aza-dC treatment, suggesting a hypermethylation mediated lossof miR-486-3p in OEC-M1 and TW2.6 (Fig. 5d, e). Furthermore, 5-Aza-dC treatment also significantly inhibited colony formation and cell proliferation in oral cancer cells (Fig. 5f, g). Collectively, these data indicate that ANK1 promoter hypermethylationis associated with miR-486-3p repression and in turn induces DDR1 expression in OSCC cells. Re-expression of miR-486-3p in oral cancer through methylation inhibition may inhibit the proliferation activity of cancer cells byimpeding DDR1 expression.

### DNMT3B involved in arecoline induced *ANK1* promoter methylation

**Fig. 6:**
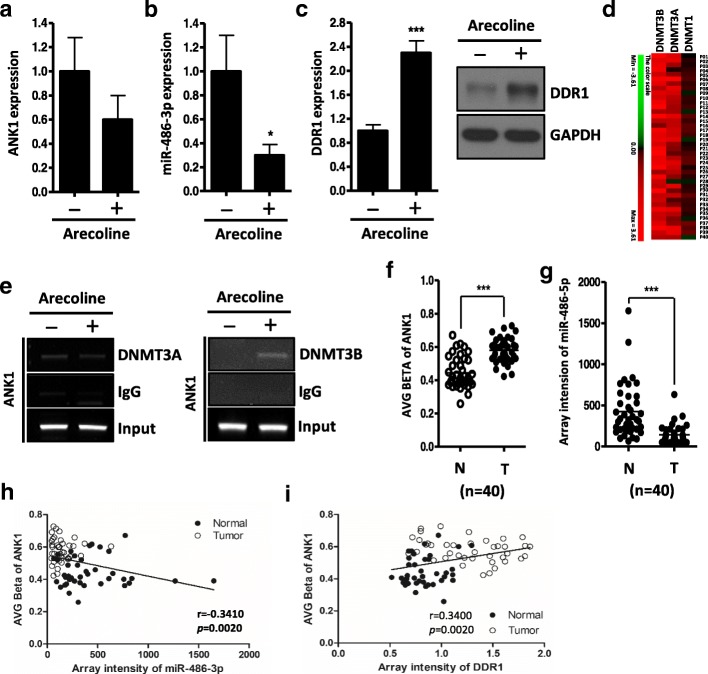
Arecoline induced DNMT3B activity and repressed ANK1 and miR-486-3p expression **a:** qRT-PCR analysis showing the expression level of ANK1 in OKF4/hTERT cells after treatment with 100 μM of arecoline for 5 days (mean ± SD). **b:** qRT-PCR analysis showing the expression level of miR-486-3p in OKF4/hTERT cells after treatment with 100 μM of arecoline for 5 days (mean ± SD; *p < 0.05). **c:** qRT-PCR (*left*) and western blot (*right*) analysis of DDR1 level in OKF4/hTERT cells after treatment with 100 μM of arecoline for 5 days. **d:** Heat map of DNMT1, DNMT3A and DNMT3B expression in 40 OSCC tissue pairs (GSE37991). Red, overexpression; green, downexpression. **e:** ChIP assay of ANK1 promoter region was performed with OKF4/hTERT cells using anti-DNMT3A antibody, anti-DNMT3B antibody or control IgG antibody as indicated. **f:** The methylation index (AVG Beta) of ANK1 promoter CpG islands from 40 matched pairs of human OSCC tissues was quantitativelydetermined using whole genome methylation data GSE38823 (mean ± SD; ***p < 0.001). **g:** Microarray intensity of miR-486-3p expression from 40 matched pairs of human OSCC tissues (mean ± SD; ***p < 0.001). **h:** Correlations between miR-486-3p expression and DNA methylation status of the ANK1 (AVG Beta) (*n* = 40). **i:** Correlations between DDR1 expression and DNA methylation status of the ANK1 (AVG Beta) (n = 40)

Betel quid chewing is one of the most important risk factor for oral cancer in Taiwan, and DNA hypermethylation has been reported to be related to betel quid chewing [37, 38]. Next, we attempted to determine the effect of arecoline, a major component of betel nut alkaloids, on the expression of ANK1 and miR-486-3p. As shown, arecoline treatment not only decreased the expression level of ANK1 mRNA and miR-486-3p (Fig. 6a, b), but also increased the DDR1 mRNA and protein level in OKF4/hTERT (an immortalized normal oral keratinocytes cells) (Fig. 6c). Furthermore, arecoline treatment increased the cellular proliferation in OKF4/hTERT cells, while knockdown of DDR1 expression blocked arecoline-induced cellular proliferation (Additional file 2: Figure S2). These results demonstrated arecoline promotes proliferation phenotypes of oral cancer cells via DDR1-dependent manner. Next, weinvestigate whether DNMTs are involved in arecoline-mediated miR-486-3p/DDR1 axis. We re-checked the 40 pairwise OSCC cDNA microarray in Fig. 1a and found that DNMT3A and 3B were dramatically up-regulated in OSCC patients (Fig. 6d). Furthermore, using chromatin immunoprecipitation (ChIP), we demonstrated that arecoline treatment increased the DNMT3B, but not DNMT3A, binding activity to ANK1 promoter region in OKF4/hTERT cells (Fig.6e). These results suggest that arecoline treatment could recruit DNMT3B binding to ANK1 promoter and caused DNA methylation. On the other hand, 5-Aza-dC treatment not only significantly rescued arecoline-repressed miR-486-3p expression, but also inhibited arecoline-induced DDR1 expression (Additional file 2: Figure S2a). Likewise, miR-486-3p mimics (PM-486-3p) treatment significantly blocked arecoline-induced DDR1 expression (Additional file 2: Figure S2b). Taken together, these studies demonstrated that miR-486-3p and its host gene*ANK1* are epigenetically repressed in oral cancer through arecoline exposure, consequently causes the DDR1 expression. Notably, miR-486-3p seems to play a key role in the arecoline-induced DDR1 expression. Further clinical analysis showed that the methylation index (AVG Beta) of ANK1 was significantly higher in oral cancer tissues compared with the normal control in our methylation array data (GSE38823) (Fig. 6f). In addition, the miR-486-3p level was significantly down-regulated in OSCC patient (Fig. 6g). Importantly, the AVG Beta of ANK1 negatively correlated with the expression of miR-486-3p (r = − 0.3410, *p* < 0.01) and positively correlated with expression of DDR1 (r = 0.3400, *p* < 0.01)(Fig. 6h, i). These results strongly imply that miR-486-3p/DDR1 axis is regulated through ANK1 promoter methylation status in OSCC patients.

## Discussion

Increased DDR1 expression has been reported and functions as an onco-protein in many types of cancer [14, 39, 40]. Avariety of oncogenic roles of DDR1 have beendescribed, including survival, proliferation, drugresistance, invasiveness, and collectivecell migration [7]. High DDR1 expression can promote survival signaling through activation of Bcl-xl, Notch1 and Ras/raf/MAPK pathway [39, 41, 42]. In the present study, we showed that DDR1 is upregulated in OSCC patients and cell lines. Knockdown of DDR1 dramatically decreases proliferation and enhances apoptosis in OSCC cells. These findings provide the framework for future studies to assess the detail mechanisms of DDR1 in OSCC survival and imply that DDR1 plays a necessary role for OSCC survival.

DDR1 has been reported to be transcriptionally regulated by p53 [42], but the *TP53* RNA level did not show significant changes in our OSCC patients microarray analysis [31]. Interestingly, several reports have shown that loss of miR-199a was found to causethe elevated expression of DDR1in hepatocellular carcinoma [20], leukemia [28], colorectal cancer [29], cutaneous squamous cell carcinoma [30], breast cancer [43] and ovarian cancer [44], suggesting that miR-199a has a critical role in DDR1 regulation. However, unlike other cancer types, miR-199a was not significantly dysregulated in our OSCC patients’ miRNA microarray data [31]. Hence, there should be other miRNAs playing the roles to regulate DDR1 function in OSCC. Herein, we demonstrate that miR-486-3p downregulated DDR1 via directly binding its 3′-UTR. Besides, the miR-486-3pexpression levels were significantly decreased in OSCC cells and OSCC tissues. Ectopic expression of miR-486-3p suppressed DDR1 expression, thereby inhibited cell proliferation and apoptosis induction. Moreover, the expression of miR-486-3pwas correlated inversely with the DDR1 expression level in 46 paired OSCC tissues (r = − 0.5838, *p* < 0.0001). Thus, considering the inverse nature of the relationship between miR-486-3p and DDR1, it seems highlylikely that miR-486-3p may act as a tumor suppressor through inhibiting DDR1 expression in OSCC. In oral tongue squamous cell carcinoma (TSCC), miR-486-3p is also significantly down-regulated and acts as a miRNA biomarker for the detection of TSCC [45]. Not only that, miR-486-3p has been also reported to be down-regulated in papillary thyroid carcinoma [46], retinoblastoma [47], cervical cancer [48], bladder cancer [49], and leukemia [50]. MiR-486-3p functions as atumor suppressor and plays a critical role in proliferationand metastasisbyrepressing various oncogenes, such as BMP2 [51], ECM1 [48], FASN and SKY [47]. Taken together, our results providea new mechanism thata low level of miR486-3p is supportive for OSCC tumorigenesis by way of DDR1 upregulation, and the clinical relevance strongly implies that this mechanism is important in OSCC.

MiR-486 is located in an intragenic region of the *ANK1* gene, and the miRNA is transcribed together with its host gene. Down regulation of miR-486-3p following ANK1 knockdown has also been reported [36]. Moreover, ANK1 transcription is associated with promoter methylation status [52, 53]. As mentioned above,the present study demonstrated that loss of ANK1 and miR-486-3p expression was significantly correlated with ANK1 promoter hypermethylation in OSCC. ANK1 and miR-486-3pcould be re-expressed by 5-Aza-dC treatment. Interestingly, aberrant promoter methylation has been reported tobe involved in oral cancer associated with betel quid chewing [37, 38]. Betel quid is the most common environmental risk factor for the development of oral cancer in Taiwan [6]. In this study, we found that arecoline treatment could recruit DNMT3B to ANK1 promoter and suppresses ANK1 and miR-486-3p expression, subsequently upregulated expression of DDR1 in oral cancer. Likewise, Tessemaet al. also showed that aberrant ANK1 methylation is highly associated with patients’ smoking history in lung cancer [53]. Based on these studiesand our findings, environmental risk factors, such as betel quid and cigarette, induced epigenetic repression of ANK1 and miR-486-3p may play important role inoral cancer development.

This study pinpoints the existence of a miR-486-3p/DDR1 targeting pathway, whichis involved in proliferation and anti-apoptosis. Thus, abnormal repression of miR486-3p in oral cancer provides a growth advantage by enhancing the tumor promoting activities of DDR1. In addition, it has been demonstrated that low expression of miR-486-3p or high expression of DDR1 are associated with a poor prognosis in various cancer types [49, 54–58]. Recently, the potent kinase inhibitory activities of imatinib, nilotinib and dasatinibmay have therapeutic implications in treatment of cancers associated with pathological DDR1 activity [59]. Interestingly, 5-fluorouraciltreatment was followed byincreased expression of miR-486-3pin esophageal adenocarcinoma and squamous cell carcinoma cell lines, possibly implicating miR-486-3pis in response to chemotherapy [60]. Therefore, more investigations are needed to explore the potential of miR-486-3p/DDR1 signaling network as noveltherapeutic targets for OSCC.

## Conclusions

**Fig. 7:**
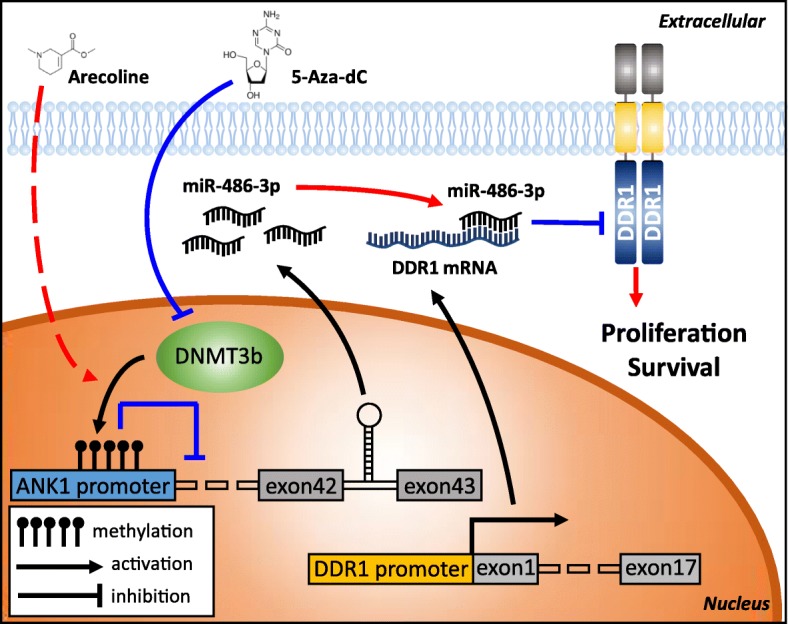
Proposed model for comprehensive oncogenic DDR1-targeting miR-486-3p and its tumor suppressor function in oral carcinogenesis

In summary, our findings highlight amiR-486-3p/DDR1 axis, the dysregulation of which leads to the proliferation and survival of oral cancer. Aberrant methylation of*ANK1* promoter is a highly prevalent in OSCC and contributes to oral carcinogenesis by epigenetic silencing of ANK1 and miR-486-3p (Fig.7). These findings may have potentially translational relevance for the development of a new targetedmolecular therapy for OSCC patients.

## Additional files

Additional file 1: **Table S1.** Clinical pathologic characteristics of the three cohorts of OSCC. **Table S2.** shRNA clone target sequence used in this study. **Table S3.** Primers sequence used in this study. **Table S4.** Correlation of the DDR1 expression with clinicopathological factors in 40 OSCC patients (*n* = 40). **Table S5.** Cox univariate and multivariate regression analysis of DDR1 and prognostic factors for overall survival in microarray cohort (*n* = 40). **Table S6.** Cox univariate and multivariate regression analysis of DDR1 and prognostic factors for overall survival in validation cohort (*n* = 46). **Table S7.** Cox univariate and multivariate regression analysis of DDR1 and prognostic factors for overall survival in survival analysis cohort (*n* = 75). (PDF 567 kb)

Additional file 2: **Figure S1.** Representative staining of DDR1 in one normal and one OSCC tissues. **Figure S2.** DDR1 knockdown inhibitsarecoline-induced cellular proliferation. **Figure S3.** Methylation inhibitor and miR-486-3p block arecoline-induced DDR1 expression. (PDF 527 kb)

## Data Availability

The dataset supporting the conclusions of this article is included within the article.

## Abbreviations

3′-UTR: 3′-Untranslated region
ChIP: Chromatin immunoprecipitation
DDR1: Discoidin domain receptor-1
HNSCC: Head and neck squamous cell carcinoma
IHC: Immunohistochemistry
miRNAs: microRNAs
MSP: Methylation specific PCR
MTT: 3-[4,5-dimethylthiazol-2-yl]-2,5-diphenyl tetrazolium bromide
OSCC: Oral squamous cell carcinoma
qPCR: Quantitative real-time PCR
RT-PCR: Reverse transcription PCR
