# Supplementary material for: MicroRNA-486-3p functions as a tumor suppressor in oral cancer by targeting DDR1
Source: J Exp Clin Cancer Res. 2019 Jun 28;38:281. doi: 10.1186/s13046-019-1283-z (PMC6599238; doi:10.1186/s13046-019-1283-z)
Supplement: Supplementary file 1 — Table S1. Clinical pathologic characteristics of the three cohorts of OSCC. Table S2. shRNA clone target sequence used in this study. Table S3. Primers sequence used in this study. Table S4. Correlation of the DDR1 expression with clinicopathological factors in 40 OSCC patients (n = 40). Table S5. Cox univariate and multivariate regression analysis of DDR1 and prognostic factors for overall survival in microarray cohort (n = 40). Table S6. Cox univariate and multivariate regression analysis of DDR1 and prognostic factors for overall survival in validation cohort (n = 46). Table S7. Cox univariate and multivariate regression analysis of DDR1 and prognostic factors for overall survival in survival analysis cohort (n = 75). (PDF 567 kb) [file 13046_2019_1283_MOESM1_ESM.pdf]

Table S1. Clinical pathologic characteristics of the three cohorts of OSCC

Characteristics	Microarray cohort (n=40)	Validation cohort (n=46)	Survival cohort (n=75)
Age			
＜60	36	35	61
≧ 60	4	11	14
T stage			
T1	1	4	6
T2	22	18	33
T3	9	9	17
T4	8	15	19
N stage			
N0	23	28	46
N1	7	8	12
N2	10	10	17
TNM stage			
I	1	3	5
II	14	10	19
III	10	11	19
IV	15	22	32
Treatment			
OP[a]	17	27	40
OP+RT[b]	17	12	23
OP+CCRT[c]	6	7	12
Perineural invasion[d]			
No	15	24	35
Yes	25	22	40
AngioLymphatic[e]			
No	22	26	41
Yes	18	20	34
Recurrence status			
No	30	30	53
Yes	10	16	22

[a]OP, operation; [b]OP+RT, operation plus radiotherapy; [c]CCRT, concurrent chemoradiotherapy; [d]perineural invasion refers to the invasion of cancer to the space surrounding a nerve refers to the invasion of cancer to the space surrounding a nerve; [e]Angiolymphatic invasion means that cancer was found in the blood vessels and lymph vessels.

Table S2. shRNA clone* target sequence used in this study.

Name	Clone ID	Target Sequence (5’-3’)
Control	TRC001	pLKO.1-base empty vector
shDDR1-D1	TRCN0000121163	TGCTGACATGAAGGGACATTT
shDDR1-A1	TRCN0000121086	GCAGGTCCACTGTAACAACAT

***Purchased from the National RNAi Core Facility (Academia Sinica, Taipei, Taiwan)**

Table S3. Primers sequence used in this study.

Primer name	Sequence (5'-3')
For gene expression	
ANK1 (F)	GGCCCGCAACGACGACACG
ANK1 (R)	AATGTGCAGGGGCGTGAATC
DDR1 (F)	CATGAGCCGGAACCTCTA
DDR1 (R)	CCACAGGGTCACACCAAA
GAPDH (F)	GAAGGTGAAGGTCGGAGT
GAPDH (R)	GAAGATGGTGATGGGATTTC
For MSP	
ANK1-M (F)	GAGCGTTCGGTTCGATAG
ANK1-M (R)	TACGAAACCTATAACGTACG
ANK1-U (F)	GGGAGTGTTTGGTTTGATAGT
ANK1-U (R)	CTCTACAAAACCTATAACATACA
For ChIP	
ANK1 (F)	CTCTTGTAATCTGCGGTCC
ANK1 (R)	GGGGCCTGTGACGTG
For miRNA	
Universal reverse	GTGCAGGGTCCGAGGT
miR-486-3p (RT)	GTTGGCTCTGGTGCAGGGTCCGAGGTATTCGCA CCAGAGC CAACATCCTG
miR-486-3p (F)	GCGGGGCAGCTCAGTA
RNU44 (RT)	GTTGGCTCTGGTGCAGGGTCCGAGGTATTCGCA CCAGAGCCAACAGTCAG
RNU44 (F)	GCCCTGGATGATGATAGCAA

F, forward; R, reverse; RT, primer sequence for reverse transcription

Table S4. Correlation of the DDR1 expression withclinicopathological factors in 40 OSCC patients (n=40)

	DDR1 RNA expression		
Characteristics	Low (n=17)	High (n=23)	*p* value[#]
Age			
Years (mean ± SD)	48±8.3	50±9.7	0.5278
Stage			0.0829
I+II	9	6	
III+IV	8	17	
Tumor status			0.1500
T1-2	12	11	
T3-4	5	12	
Lymph node status			**0.0369***
N0	13	10	
N1-N3	4	13	
Perineural invasion[a]			**0.0166***
No	10	5	
Yes	7	18	
AngioLymphatic[b]			**0.0091****
No	13	8	
Yes	4	15	
Recurrence status			0.3558
No	14	16	
Yes	3	7	

[#]*p* value for age was derived from a two-tailed Student's *t* test. SD represents standard deviation; other *p* values were derived with a two-tailed Pearson chi-square test. *$p<$ 0.05; **$p<$ 0.01.

[a]perineural invasion refers to the invasion of cancer to the space surrounding a nerve refers to the invasion of cancer to the space surrounding a nerve; [b]Angiolymphatic invasion means that cancer was found in the blood vessels and lymph vessels.

Table S5. Cox univariate and multivariate regression analysis of DDR1 and prognostic factors for overall survival in microarray cohort (n=40)

Variables	Univariate		Multivariate	
	coefficient	p-value	coefficient	p-value
Age	0.01506	0.62	0.02408	0.49
TNM stage (I+II *vs.* III+ IV)	0.4372	0.46	0.64728	0.377
Treatment (Op only *vs.* Op +)	-0.09291	0.863	-0.39764	0.515
DDR1	0.5534	0.463	0.37662	0.665

Op +, Op +RT or Op + CCRT

Table S5. Cox univariate and multivariate regression analysis of DDR1 and prognostic factors for overall survival in validation cohort (n=46)

Variables	Univariate		Multivariate	
	coefficient	p-value	coefficient	p-value
Age	-0.0056	0.823	-0.01186	0.658
TNM stage (I+II *vs.* III+ IV)	0.5116	0.367	0.39174	0.578
Treatment (Op only *vs.* Op +)	0.4730	0.317	0.30965	0.594
DDR1	-0.0085	0.984	-0.01825	0.968

Op +, Op +RT or Op + CCRT

Table S7. Cox univariate and multivariate regression analysis of DDR1 and prognostic factors for overall survival in survival analysis cohort (n=75)

Variables	Univariate		Multivariate	
	coefficient	p-value	coefficient	p-value
Age	0.0171	0.403	0.007767	0.718
TNM stage (I+II *vs.* III+ IV)	0.6729	0.149	0.834273	0.1236
Treatment (Op only *vs.* Op +)	0.1877	0.632	-0.299162	0.5128
DDR1 (low *vs.* high)	0.8499	**0.0307***	0.90453	**0.0273***

Op +, Op +RT or Op + CCRT

***Two-sided Cox proportional hazards regression using normal approximation and $p < 0.05$ was considered statistically significant.**
