# Supplementary material for: MicroRNA-486-3p functions as a tumor suppressor in oral cancer by targeting DDR1
Source: J Exp Clin Cancer Res. 2019 Jun 28;38:281. doi: 10.1186/s13046-019-1283-z (PMC6599238; doi:10.1186/s13046-019-1283-z)

Additional file 2: Fig. S1. Representative staining of DDR1 in one normal and one OSCC tissues.

a: Tissue microarray analysis of 45 OSCC and six normal specimens (OR2081, HN811, BC34011) purchased from Biomax Inc. (Rockville, MD). The expression level for DDR1 was assessed by distribution score and staining score. Distribution score was defined as the percentages of positive cells: 1 (<10%), 2 (10–50%), and 3 (>50%). Staining score was defined as 1+ (mild), 2+ (moderate), and 3+ (intense). **b:** A final score was obtained by multiplying the distribution score and intensity score. 0–3 was defined as "low" and 4-9 was defined as "high" expression. Two pathologists independently assessed all the scorings. Subcellular localization, distribution score and intensity score are denoted in parentheses. Summary of high (score ≥ 4) staining of DDR1 are shown in the indicated tissues. The associations between DDR1 and tumor grade were analyzed by Fisher's exact test.

Additional file 2: Fig. S2. DDR1 knockdown inhibitsarecoline-induced cellular proliferation.

a: Western blot analysis of DDR1 level in OKF4/hTERT cells after treatment with shRNA control plasmid (TRC001), or arecoline (100 µM) plus TRC001, orarecoline(100 µM) plus DDR1 knockdown plasmid (shDDR1-A1) for 5 days. GAPDH was used as an internal control.**b:**Growth rates of OKF4/hTERT cells after treatment with shRNA control plasmid (TRC001), or arecoline (100 µM) plus TRC001, orarecoline (100 µM) plus DDR1 knockdown plasmid (shDDR1-A1) for indicated days. All data are presented as mean ± SD; ***p< 0.001.

Additional file 2: Fig. S3. Methylation inhibitor and miR-486-3p block arecoline-induced DDR1 expression.

a: Western blot analysis of DDR1 level in OKF4/hTERT cells after treatment with arecoline (100 µM) alone or combined with 5-Aza-dC (5 µM) for 5 days (*upper*). qRT-PCR analysis showing the expression

level of miR-483-5p in OKF4/hTERT cells after treatment with arecoline (100 µM) alone or combined with 5-Aza-dC (5 µM) for 5 days (*lower*). **b:** Western blot (*upper*) and qRT-PCR (*lower*)analysis of DDR1 level in OKF4/hTERT cells after treatment with control mimics (NC), or arecoline (100 µM) plus control mimics (NC), orarecoline (100 µM) plus miR-486-3p mimics (PM-486-3p, 20 nM) for 5 days. GAPDH was used as an internal control.All data are presented as mean ± SD; ** $p < 0.01$; *** $p < 0.001$.

Additional file 2: Fig. S1.

a

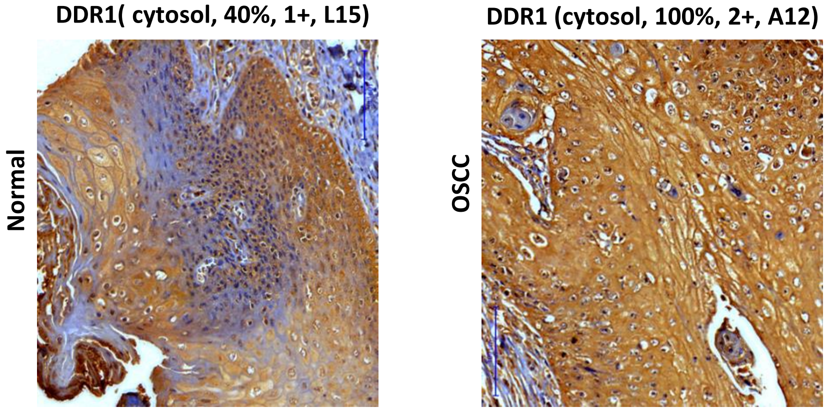

b

	Samples	High DDR1
Normal	6	0
OSCC grade 1	33	23 ($p < 0.01$)
OSCC grade 2-3	12	10 ($p < 0.01$)

Additional file 2: Fig. S2.

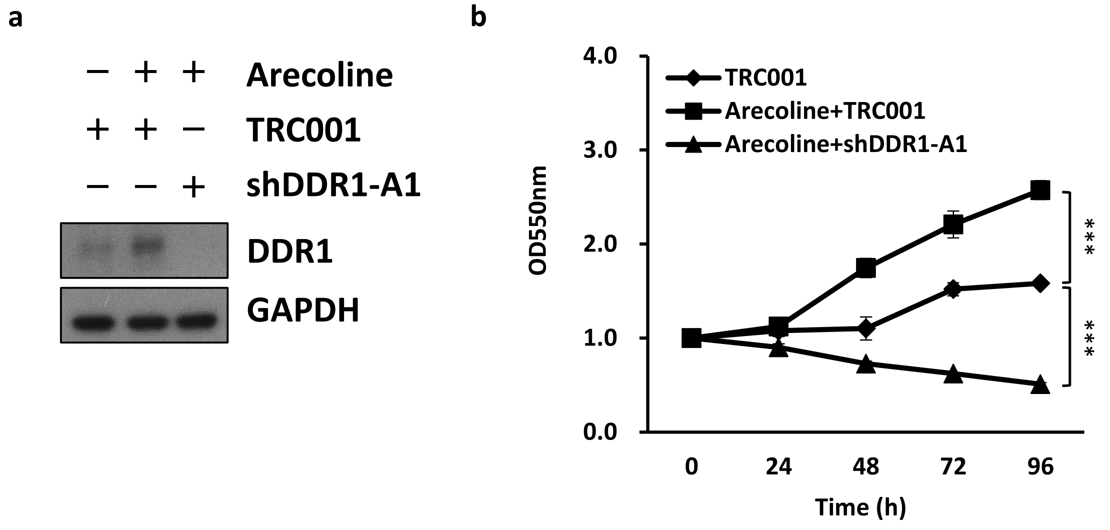

Additional file 2: Fig. S3.

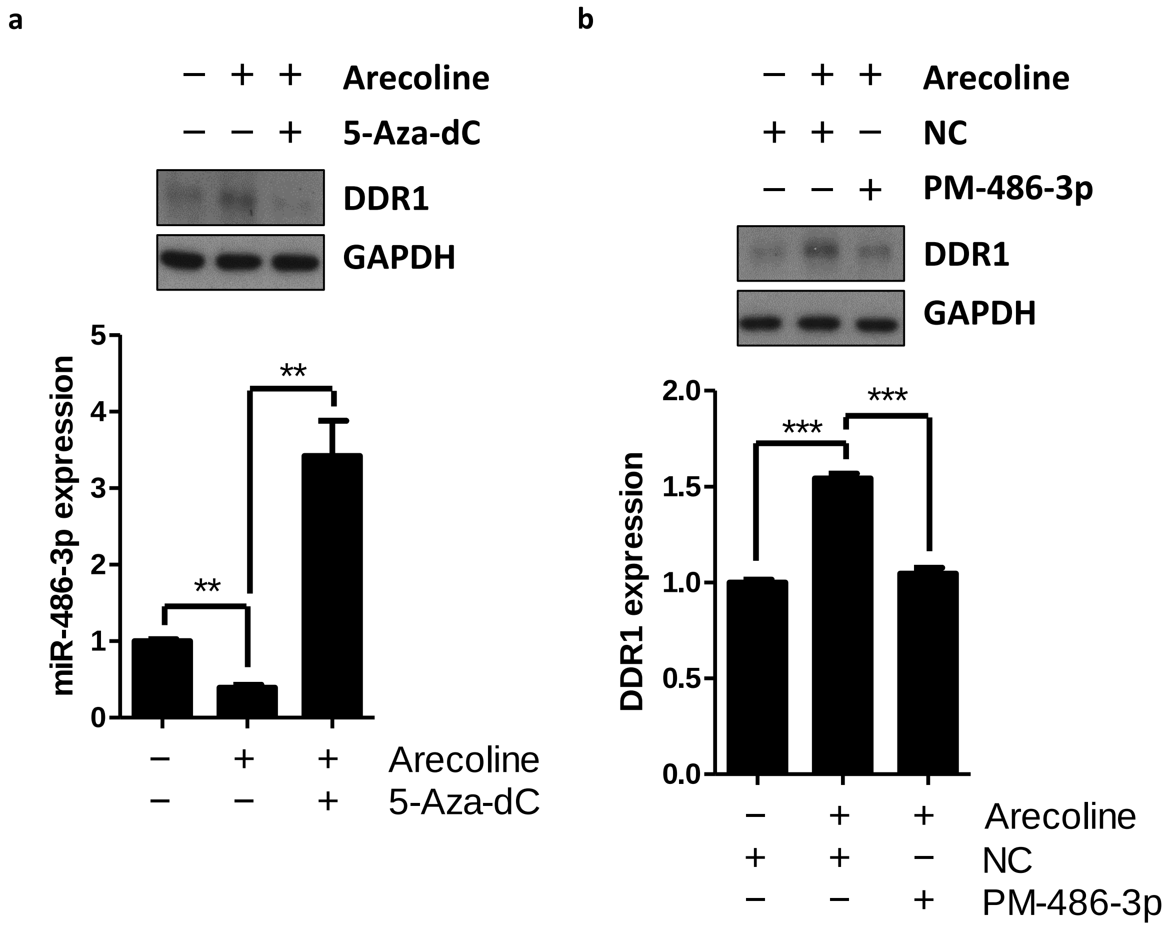

Supplement: Supplementary file 2 — Figure S1. Representative staining of DDR1 in one normal and one OSCC tissues. Figure S2. DDR1 knockdown inhibitsarecoline-induced cellular proliferation. Figure S3. Methylation inhibitor and miR-486-3p block arecoline-induced DDR1 expression. (PDF 527 kb) [file 13046_2019_1283_MOESM2_ESM.pdf]
